# Parabens at Environmental Levels Modulate Virulence and Antimicrobial Tolerance of Exposed Biofilm Cells

**DOI:** 10.3390/antibiotics15060565

**Published:** 2026-06-01

**Authors:** Ana Rita Pereira, Manuel Simões, Inês B. Gomes

**Affiliations:** LEPABE (Laboratory for Process Engineering, Environment, Biotechnology and Energy), ALICE (Associate Laboratory in Chemical Engineering), Faculty of Engineering, University of Porto, Rua Dr. Roberto Frias, 4200-465 Porto, Portugal; anaritafsp@fe.up.pt (A.R.P.); mvs@fe.up.pt (M.S.)

**Keywords:** antimicrobial tolerance, biofilms, drinking water, motility, parabens, virulence factors

## Abstract

**Background/Objectives**: Parabens are widely used preservatives detected at trace levels in drinking water. Although their endocrine-disrupting effects are well established, their long-term impact on environmental bacteria remains poorly understood. This study investigated the effects of parabens on changes in bacterial phenotypic virulence traits and antimicrobial tolerance of bacteria within drinking water biofilms. **Methods**: *Acinetobacter calcoaceticus* and *Stenotrophomonas maltophilia* biofilms were grown on polyvinyl chloride coupons for 26 days under exposure to methyl- (MP), propyl- (PP), butyl-paraben (BP), or a paraben mixture (MIX) at 0.15 µg/L. Biofilm regrowth and virulence-associated traits, including motility (swimming, swarming, and twitching), extracellular enzymes (gelatinase, protease, and lipase), and siderophore production, were evaluated. The effect of prolonged MP exposure (10 weeks) on antimicrobial tolerance was assessed. **Results**: In *A. calcoaceticus*, MP reduced biofilm biomass by 32%, whereas MIX increased biomass by 25% and culturability (1.1-fold). *S. maltophilia* showed increased biofilm culturability with PP (50%), and increased biomass of 2.6-, 2.4-, and 1.8-fold for PP, BP, and MIX, respectively. Biofilm cells exhibited higher virulence factor production than planktonic counterparts. *S. maltophilia* biofilm cells exposed to BP and MIX showed enhanced swimming and swarming motility, with halo diameters up to fivefold larger than controls. Lipase production increased under BP and MIX exposure, whereas MP exposure reduced it. A MP-induced reduction in motility was observed for *A. calcoaceticus* and *S. maltophilia*. Long-term MP exposure results in reduced susceptibility to ceftazidime and minocycline in *A. calcoaceticus*. **Conclusions**: Environmentally relevant concentrations of parabens can modulate bacterial virulence traits, increasing biofilm formation, motility and lipase production, and antimicrobial tolerance.

## 1. Introduction

Despite the application of multiple and highly effective disinfection processes, drinking water (DW) is not sterile. Water reaching consumers’ taps may contain between 10^3^ and 10^6^ cells mL^−1^ [1]. In most cases, the presence of these microbial cells does not pose a health concern. However, the formation of biofilms within DW distribution systems (DWDS) and the potential release of pathogenic bacteria into the bulk water can compromise water quality and safety. The occurrence of DW-associated outbreaks worldwide underscores the critical importance of investigating DW microbial communities, particularly their virulence potential, to improve our understanding of how outbreaks emerge and develop.

A recent publication describes DW-associated outbreaks reported in the United States between 2015 and 2020. During this period, 214 outbreaks linked to DW were documented. Of these, 87% were associated with biofilms, and 80% were linked to water from public water systems. Overall, these outbreaks resulted in at least 2140 cases of illness, 563 hospitalizations (26% of cases), and 88 deaths (4% of cases) [2]. These data highlight the potential impacts of biofilm formation and bacterial proliferation within DWDS, even in a developed and industrialized country. The situation in developing countries is likely to be even more concerning, with the potential for substantially higher numbers of cases, hospitalizations, and deaths due to additional challenges in water treatment and infrastructure [3].

Therefore, studying the mechanisms, such as virulence factors production, that enable bacteria to survive in DWDS and infect hosts is of utmost importance. Virulence factors are bacterial traits that allow microorganisms to overcome host defenses and facilitate invasion and infection [4]. These factors include, for example, siderophores—molecules that chelate ferric iron (Fe^3+^) from the surrounding environment, making it available to the producing microorganism—as well as enzymes such as proteases, lipases, and mucinases, which can degrade or weaken host tissues [5]. In DWDS, these factors enable bacteria to form biofilms, resist disinfection, scavenge nutrients (especially iron), and survive in the environment before potentially infecting the DW consumer.

Several studies have highlighted the virulence profiles of bacteria isolated from DW. Wei et al. [6] collected *Pseudomonas aeruginosa* from 23 Chinese cities over the course of a year, obtaining 132 isolates, all of which carried one or more virulence genes. Similarly, Gomez-Alvarez et al. [7] reported the presence of DNA sequences associated with virulence factors in the microbial community of chlorine-treated DW. Even in bottled DW, genes related to virulence, as well as antibiotic resistance genes (ARGs), have been detected, underscoring the potential for pathogenic traits to persist in treated water sources.

In addition to these microbiological concerns, DW quality can also be compromised by chemical contaminants, including micropollutants such as parabens—a class of preservative compounds widely used in personal care products, pharmaceuticals, and food. Parabens have been detected worldwide in DW at trace concentrations ranging from ng/L–µg/L, reflecting their incomplete removal during wastewater and DW treatment processes [8]. The simultaneous presence of biofilms and parabens may significantly alter microbial behavior and compromise water safety. Recent studies have demonstrated that the presence of parabens in DWDS can significantly alter bacterial behavior and biofilm dynamics. Exposure to environmentally relevant concentrations of parabens, specifically methylparaben (MP), has been shown to increase biofilm proliferation, including higher cellular density, thickness, and viability compared with non-exposed biofilms [5].

Importantly, MP exposure has been reported to compromise the efficacy of chlorine-based disinfection, with parabens-exposed biofilms displaying greater tolerance to free chlorine than non-exposed counterparts, reducing the efficiency of DW treatment. Moreover, parabens exposure has been associated with increased bacterial tolerance to antibiotics and potential shifts in microbial community resistance profiles, suggesting that these contaminants may contribute to the dissemination of antimicrobial resistance in aquatic environments.

Although these findings suggest that parabens may influence bacterial adaptation and survival within DWDS, their potential effects on bacterial virulence remain poorly understood.

Therefore, this study investigated the influence of environmentally relevant concentrations of parabens on the persistence and phenotypic expression of virulence of DW bacteria growing as biofilms within DWDS. Specifically, the effects of different parabens—methylparaben (MP), propylparaben (PP), butylparaben (BP), and a paraben mixture (MIX)—on biofilm regrowth, bacterial motility (swimming, swarming, and twitching), production of extracellular enzymes and siderophores, and bacterial susceptibility to antibiotics, were evaluated. Importantly, unlike previous studies that focused exclusively on short-term exposure to MP, the present work assessed bacterial adaptation after long-term biofilm exposure (26 d), better reflecting chronic environmental contamination scenarios. Moreover, this is the first study to perform an integrated phenotypic assessment of functional adaptations occurring under chronic exposure conditions by combining long-term paraben exposure, virulence-related phenotypes, and antimicrobial tolerance in DW biofilms exposed to multiple parabens varying the alkyl chain, and their mixture. Understanding these effects is essential for improving risk assessment and supporting the development of strategies to mitigate potential consequences on human health associated with DW biofilms.

## 2. Results

### 2.1. Impact of 26-Day Paraben Exposure on Bacterial Biofilm Formation

The characterization of single-species biofilms of *A. calcoaceticus* and *S. maltophilia* grown on polyvinyl chloride (PVC) coupons for 26 days with the continuous exposure to the selected parabens—MP, propylparaben (PP), butylparaben (BP), and their mixture (MIX)—at 0.15 µg/L is detailed in Pereira et al. [5]. Information about the number of culturable cells and total density of biofilms is presented in Supplementary Material Figure S1.

**Figure 1:**
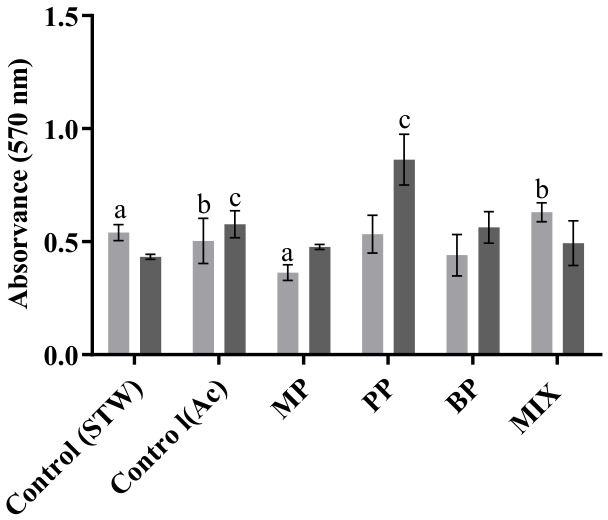
Biofilm-forming ability expressed as total biomass of *A. calcoaceticus* (■) and *S. maltophilia* (■) cells derived from 26-day-old biofilms exposed and non-exposed to parabens at 0.15 µg/L. ^a, b, c^—Correspond to conditions that have statistically significant differences from the respective control (ANOVA, post hoc Tukey’s test, *p* < 0.05).

The bacterial ability to form biofilms after exposure to parabens at 0.15 µg/L for 26 days was evaluated in terms of total biomass and culturability. The total biomass was quantified using the crystal violet (CV) quantitative assay (Figure 1). For *A. calcoaceticus*, the exposure to MP reduced biomass production by 32%. In contrast, exposure to the MIX solution led to a significant increase of 25% in comparison to the respective control (*p* < 0.05, Figure 1). *S. maltophilia* also exhibited a stimulatory response under PP exposure, with biofilm biomass increase up to 50% relative to the control (*p* < 0.05, Figure 1). Biofilms exposed to BP and to the MIX formulation exhibited biomass values comparable to the control (Ac), indicating no significant enhancement of total biofilm biomass.

**Figure 2:**
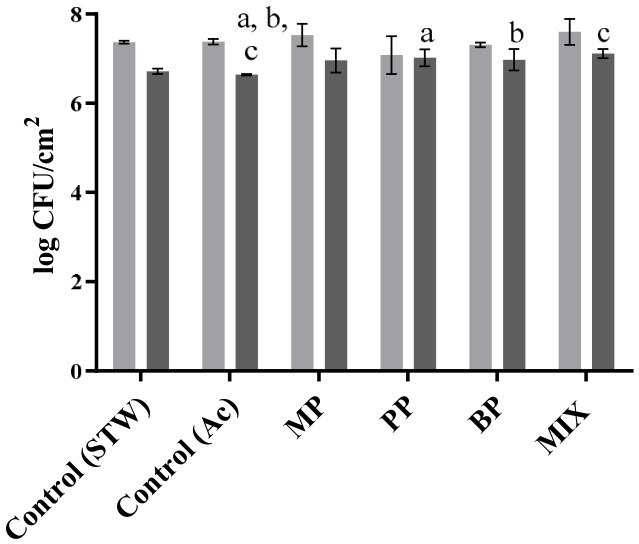
Biofilm-forming ability in terms of culturable cells—log CFU/cm^2^ of *A. calcoaceticus* (■) and *S. maltophilia* (■) cells derived from 26-day-old biofilms exposed and non-exposed to parabens at 0.15 µg/L. ^a, b, c^—Correspond to conditions that have statistically significant differences from the respective control (ANOVA, post hoc Tukey’s test, *p* < 0.05).

Biofilm culturability, expressed as log colony-forming units (CFU)/cm^2^, showed distinct responses regarding bacterial species and parabens (Figure 2). However, in *A. calcoaceticus*, paraben exposure did not significantly affect bacterial culturability (*p* > 0.05; Figure 2). Likewise, in *S. maltophilia*, MP exposure resulted in no significant difference in culturability compared with the control (STW). In contrast, exposure to PP, BP, and the MIX formulation resulted in significant increases in culturable cells, corresponding to 2.6-, 2.4-, and 1.8-fold increases, respectively, relative to the control (Ac) (*p* < 0.05; Figure 2). Therefore, PP exposure seems to effectively potentiate the growth of *S. maltophilia* biofilms, as further confirmed by the increased biomass production.

Overall, these results demonstrate that the effects of parabens on biofilm formation are compound- and species-dependent. PP and the MIX formulation exerted the strongest stimulatory effects on biofilm formation reflected by increases in both biofilm biomass production and culturability for *S. maltophilia* and *A. calcoaceticus*, respectively.

### 2.2. Evaluation of Parabens on the Production of Virulence Factors

The effects of MP, PP, BP, and MIX exposure at 0.15 µg/L on the production of virulence factors by bacterial cells (*A. calcoaceticus* and *S. maltophilia*) from single-species biofilms grown on PVC for 26 days were also evaluated, as described in Section 4.4. An overall increase in the production of virulence factors of both bacteria from biofilms (Table 1 and Table 2) in comparison to their planktonic counterparts (Supplementary Material Table S1) was observed. In *A. calcoaceticus*, planktonic cells did not produce siderophores or gelatinase, whereas 26-day biofilm cells exhibited clear production of both, as indicated by halo formation (Table S1). Protease production was also approximately three-fold higher in biofilm cells than in planktonic cells (*p* < 0.05, Table 1). Regarding the effects of parabens on *A. calcoaceticus* motility, only MP was able to significantly decrease swimming, swarming and twitching motility in comparison to the control (Table 1, *p* < 0.05). In contrast, no significant changes caused by parabens exposure on extracellular enzyme production or siderophore synthesis by *A. calcoaceticus*, were observed (*p* > 0.05).

In *S. maltophilia*, biofilm cells exhibited increased siderophore (1.7-fold) and lipase production (2.3-fold) relative to planktonic cells, while protease production decreased upon transition to the biofilm lifestyle (*p* < 0.05, Table 2). This bacterium reported changes in motility under parabens exposure. In particular, MP led to a marked reduction in swimming and swarming motilities, with halo diameters decreasing to about half of those measured for non-exposed biofilm cells (*p* < 0.05, Table 2). In contrast, exposure to BP and the MIX formulation for 26 days significantly enhanced swimming and swarming *S. maltophilia* motility. Specifically, swimming motility increased by 2.5- and 4.5-fold in BP- and MIX-exposed biofilm cells, respectively, compared to non-exposed counterparts (*p* < 0.05). Swarming was also strongly potentiated by BP and MIX, with exposed cells exhibiting halos up to five times larger than those of non-exposed biofilm cells (*p* < 0.05, Table 2). In addition to motility changes, exposure to MP resulted in a reduction in lipase production to approximately half of control levels, while BP and MIX exposure led to a twofold increase in lipase production (*p* < 0.05).

### 2.3. Tolerance to Antibiotics After Long Exposure to Methylparaben at 0.15 µg/L

**Figure 3:**
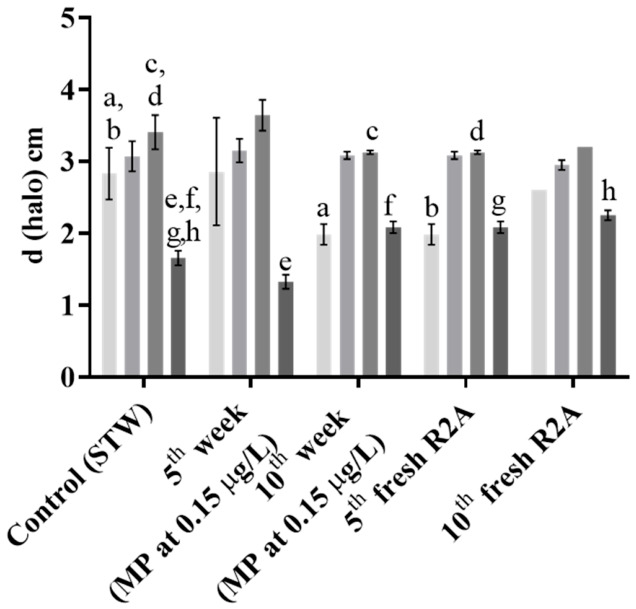
Diameter (d) of the inhibition halos (cm) of *A. calcoaceticus* exposed to MP at 0.15 µg/L after 5 and 10 weeks, and grown on fresh R2A without MP presence for an additional 5 and 10 weeks against CEF ■, LEV ■, MINO ■, and TMP-SMX ■. ^a, b, c, d, e, f, g, h^—Correspond to conditions that have statistically significant differences from the respective control (*t*-test, *p* < 0.05).

**Figure 4:**
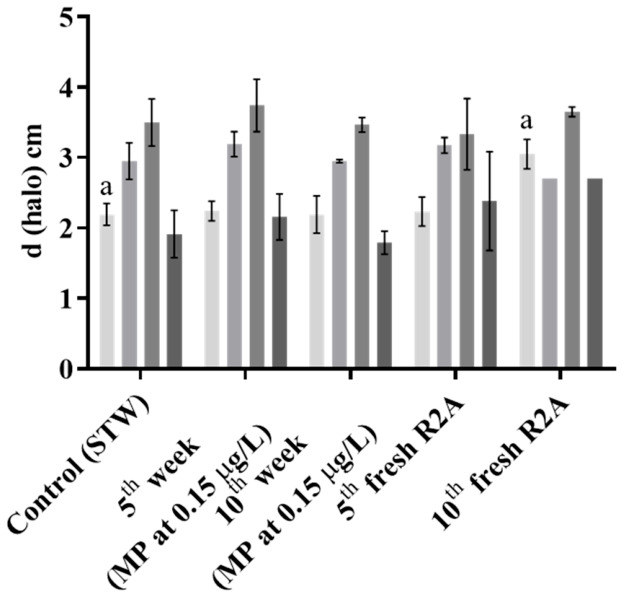
Diameter (d) of the inhibition halos (cm) of *S. maltophilia* exposed to MP at 0.15 µg/L after 5 and 10 weeks, and grown on fresh R2A without MP presence for an additional 5 and 10 weeks against CEF ■, LEV ■, MINO ■, and TMP-SMX ■. ^a^—Correspond to conditions that have statistically significant differences from the respective control (*t*-test, *p* < 0.05).

Both planktonic bacteria were grown on R2A agar without MP, supplemented with MP at 0.15 µg/L for 10 weeks, and then grown on fresh R2A without MP presence for another 10 weeks. The susceptibility to antibiotics was evaluated every 5 weeks, and the obtained inhibition halo diameters are presented in Figure 3 and Figure 4. Data showed that after 5 weeks of MP exposure at 0.15 µg/L, *A. calcoaceticus* were less susceptible to trimethoprim-sulfamethoxazole (TMP-SMX) in comparison to non-exposed bacteria (*p* < 0.05). However, after additional 5 weeks of exposure to MP, *A. calcoaceticus* became more susceptible to TMP-SMX, and this increased susceptibility was maintained even without MP presence for more 10 weeks (Figure 3). In contrast, reduced *A. calcoaceticus* susceptibility to ceftazidime (CEF) and minocycline (MINO) was observed after 10 weeks of MP exposure at 0.15 µg/L, as evidenced by smaller inhibition zone diameters. This reduced susceptibility was maintained after 5 weeks of bacterial growth in fresh R2A medium in the absence of MP presence (*p* < 0.05, Figure 3).

Curiously, the exposure to MP at 0.15 µg/L for 5 and even 10 weeks did not cause significant changes in *S. maltophilia* susceptibility to all tested antibiotics. However, an increase in *S. maltophilia* susceptibility to CEF was verified after 10 weeks of passages in fresh R2A (*p* < 0.05, Figure 4).

## 3. Discussion

Parabens in cosmetics, pharmaceuticals, and food are primarily regulated based on their endocrine-disrupting effects in organisms such as aquatic animals and humans [9,10]. This study provides further insight into how chronic exposure to trace concentrations of parabens representative of those detected in DW systems [8,11,12] can act as a selective pressure, shaping microbial ecology within DWDS. By evaluating long-term (26 days) biofilm exposure to different parabens and their mixture, this work provides new insights into how these contaminants influence biofilm persistence, virulence-associated traits (bacterial motility, extracellular enzymes, and siderophores production), and antibiotic tolerance, contributing to a more comprehensive understanding of the ecological implications of contaminant-driven biofilm adaptation in DW systems.

Results showed that DW bacteria grown under parabens exposure at environmentally relevant levels showed increased potential of biofilm formation, accompanied by an increase in the production of extracellular enzymes and increased motility. All together these observations highlight an enhanced bacterial proliferation and emphasize the bacterial modulation of phenotypic properties under chronic exposure to parabens. Increased bacterial tolerance to antibiotics under these conditions was also verified, emphasizing the ability of environmental contaminants to modulate bacterial response to stress conditions. However, the responses vary between bacteria (*A. calcoaceticus* and *S. maltophilia*), highlighting species-specific adaptive strategies. The increase in biofilm biomass and culturability observed in *A. calcoaceticus* following MIX exposure, and for *S. maltophilia* following PP exposure is consistent with previous studies reporting stress-adaptive responses induced by environmental concentrations of parabens that favor biofilm persistence [5]. The increased *S. maltophilia* biomass production observed due to BP and MIX exposure also highlights the adaptability of this bacterium to chemical stressors and its capacity to exploit sub-inhibitory concentrations of antimicrobials and disinfectants to enhance biofilm formation [13,14]. The stronger effects observed for the more hydrophobic parabens further indicate that physicochemical properties linked to paraben alkyl chain length significantly influence bacterial responses, potentially through interactions with cell membranes or biofilm extracellular polymeric substances (EPSs) [15,16,17]. Indeed, confocal laser scanning microscopy analyses have previously shown that MP exposure increased extracellular polysaccharides production [18]. However, these studies were conducted under different experimental conditions, including dual-species biofilm models, distinct polymeric surfaces for biofilm formation, shorter exposure periods (7 days), and higher MP concentrations (15 µg/L). Despite these methodological differences, the observed responses suggest that phenotypic adaptation may occur even at higher paraben concentrations and under relatively short exposure periods.

Across both species, biofilm-derived cells exhibited higher virulence-associated production than their planktonic counterparts, reinforcing the well-established concept that biofilms serve as reservoirs of pathogens in DW systems [19,20]. Bacterial motility is widely recognized as an important virulence-associated trait, as it facilitates surface colonization, biofilm development, environmental dissemination, and host interaction [21,22].

Motility, such as swimming and swarming, is primarily mediated by flagella, which enable bacteria to actively migrate toward favorable niches, penetrate host tissues, and overcome physical barriers [23]. Flagella have also been shown to function as virulence factors themselves, contributing to adhesion, immune evasion, and host cell stimulation. *S. maltophilia* is a flagellated bacterium, typically possessing a single polar flagellum that supports swimming and swarming motility and contributes to surface colonization and virulence-related behaviors [13]. Flagellar motility in *S. maltophilia* has been associated with biofilm formation, environmental persistence, and host interaction [13]. In contrast, *A. calcoaceticus*, like other members of the *Acinetobacter* genus, is generally considered non-flagellated and lacks flagellum-mediated motility [24,25]. Instead, its surface-associated movement is primarily driven by type IV pili, which enable twitching motility and contribute to microcolony formation, as well as the early stages of biofilm establishment and persistence [25]. This distinction highlights that motility-associated virulence in DW bacteria can be mediated by different cellular structures, depending on species-specific physiological traits.

Notably, BP and MIX exposure at 0.15 µg/L for 7 days markedly enhanced swimming and swarming motility in *S. maltophilia*, which may help explain the increased biofilm formation ability observed under paraben exposure, as enhanced motility is associated with improved surface colonization. Arruda et al. [26] also reported increased motility for *S. maltophilia* under musk fragrances exposure at 0.15 µg/L, corresponding to the maximum concentration of musk compounds detected in DW. In DW systems, increased motility may further promote bacterial dissemination within distribution networks and facilitate transitions between planktonic and biofilm lifestyles, reinforcing the role of motility as a critical determinant of bacterial pathogenicity [27]. In contrast, MP exposure consistently reduced swimming motility in *S. maltophilia* and twitching motility in *A. calcoaceticus*, indicating that different parabens may induce distinct effects on bacterial motility traits. Thiroux et al. [28] also reported reduced swimming and swarming motilities in *Pseudomonas aeruginosa* following exposure to MP and EP at concentrations up to 100 µM, further supporting the species-specific effects of parabens on bacterial motility. The observed changes in motility may reflect alterations in the regulation or functionality of motility-associated structures, including flagella and type IV pili; however, molecular analyses would be required to confirm these mechanisms [21,22]. Similar reductions in bacterial motility have also been reported following exposure to subinhibitory concentrations of pollutants. For example, reduced motility was observed in *Rhodanobacter* under metal-contaminated conditions [29], and in *Paramecium* exposed to erythromycin at concentrations commonly detected in DW [30]. Moreover, a previous study assessing the effect of MP on DW bacterial motility over a shorter exposure period (7 days) using multiple polymeric materials for biofilm formation reported enhanced swimming motility in *S. maltophilia* [5]. These observations suggest that exposure duration is a critical factor influencing bacterial responses to MP, potentially leading to distinct adaptive outcomes over time.

Regarding extracellular enzymes production, the concomitant increase in lipase production under BP and MIX exposure verified for *S. maltophilia* further supports the notion that paraben exposure may promote traits associated with nutrient acquisition and virulence [31]. Indeed, lipases facilitate lipid degradation for nutrient acquisition, disrupt host cell membranes, and promote tissue invasion and biofilm persistence [31]. Gelatinase and overall protease production seemed not to be significantly affected by the selected parabens; however, studies on environmental contaminants (e.g., pharmaceuticals and metals) suggest that chemical stress can effectively stimulate bacterial adaptive responses, including the production of extracellular enzymes that potentiate bacterial pathogenicity [32]. For example, Wang et al. [33] reported that trace levels of sulfadiazine and ciprofloxacin in DWDS increased bacterial protease production, suggesting a stress-induced upregulation of virulence-associated enzymes. In contrast, reduced protease production has been observed following exposure to di-n-butyl phthalate and metallic nanoparticles [34,35]. Enhanced gelatinase production was verified in bacteria from heavy-metal-polluted [36] and MP-contaminated aquatic environments [5]. These studies underscore that extracellular enzyme production is a dynamic and responsive trait, modulated by environmental stressors in ways that may enhance bacterial persistence and resistance under certain stress conditions. However, the observed phenotypic responses are interpreted at the functional level, and further studies incorporating targeted gene expression analyses will be required to elucidate the underlying molecular pathways involved in paraben-induced modulation of bacterial motility and extracellular enzyme production.

In sum, distinct virulence factor responses (motility and lipases production) were observed depending on both the bacterial species and the paraben tested. In *S. maltophilia*, MP exposure consistently reduced motility and lipase production, whereas BP and MIX solutions promoted increased motility and lipase production. PP showed little or no effect. Interestingly, this pattern suggests that the bacterial response may be influenced by physicochemical properties associated with paraben alkyl chain length. In contrast, *A. calcoaceticus* exhibited a more limited response profile, with motility reduction being observed only in some conditions following MP exposure, while no significant effects were detected for PP, BP, or MIX. These findings indicate that paraben-induced motility adaptation and lipase production may be species-dependent and influenced by the chemical characteristics of individual parabens.

Prolonged MP exposure at 0.15 µg/L for 10 weeks was associated with reduced susceptibility of *A. calcoaceticus* to diverse antibiotics. This observation is relevant, as decreased susceptibility to antibiotics such as CEF and MINO may contribute to enhanced bacterial persistence under treatment pressure. Similar adaptive responses have been reported following chronic exposure to disinfectants and pharmaceutical residues in DW environments, where long-term exposure to sub-inhibitory chemical stress may favor the emergence of more tolerant phenotypes [37]. Although the molecular mechanisms underlying these responses were not investigated in the present study, previous studies have suggested that chronic exposure to antimicrobial or chemical stressors may affect bacterial physiological and adaptive responses through multiple pathways [38,39,40]. Increasing evidence suggests that parabens may contribute to the dissemination of antimicrobial resistance determinants in aquatic environments. For example, exposure to mixtures of antibiotics and parabens has been associated with increased abundances of *sul1*, *sul2* and *intI1*, indicating enhanced potential for sulfonamide resistance and horizontal gene transfer [39]. Likewise, long-term exposure (15 weeks) to parabens (MP, EP, PP, and BP) at 90 µg/L has been shown to increase the abundance of tetracycline- and sulfamethoxazole-resistant bacteria in freshwater microbial communities, with effects particularly pronounced for lower-molecular-weight parabens such as MP [40]. The presence of ARGs, including efflux pump- and erythromycin resistance-associated determinants, has also been linked to MP (1970 ng/g dust) contamination in indoor dust microbiomes [41]. More recently, MP exposure has been shown to promote ARG dissemination in sludge systems, particularly when combined with biocides, further highlighting MP’s potential role in resistance propagation [42]. Although antimicrobial resistance determinants were not evaluated in the present study, these previous findings support the hypothesis that chronic exposure to parabens may influence bacterial adaptive responses and antibiotic tolerance under environmentally relevant conditions.

Curiously, a consistent linear relationship between paraben exposure duration or concentration and increased antibiotic resistance is not observed. Indeed, a recent study showed that exposure of greywater bacteria to PP (up to 0.2 mg/L) increased antibiotic resistance after two weeks; however, this effect did not further intensify after four weeks of exposure, and the abundance of ARGs was not proportional to micropollutant concentration [43]. Overall, these findings suggest that parabens may influence bacterial antimicrobial susceptibility under certain exposure conditions. However, future research should aim to elucidate the molecular mechanisms underlying the effects of parabens at sub-inhibitory concentrations on changing bacterial susceptibility to antibiotics. In parallel, future studies should also investigate the impact of parabens in real or more complex DW biofilms, to validate and strengthen the ecological relevance of the present findings and better reflect conditions in DWDS.

From a One Health perspective, these results are noteworthy because DW biofilms represent a critical interface between environmental contamination and human exposure. The results indicate that parabens can modulate biofilm regrowth, enhance bacterial motility and extracellular enzyme production as virulence-related traits, and increase antimicrobial tolerance, collectively suggesting a potential rise in microbial risks in DWDS. For example, a recent study reported that MP exposure may increase DW bacterial invasion into human gingival fibroblasts, potentially elevating the risk of human infection [44]. Therefore, the health of DW consumers may be affected by altered microbial phenotypes within DWDS. In this context, future regulatory frameworks for DW safety could consider monitoring micropollutants such as parabens alongside health risks.

## 4. Materials and Methods

### 4.1. Parabens Solutions

Parabens including MP, PP and BP (Sigma-Aldrich, Steinheim, Germany) were selected as environmental contaminants due to their frequent detection in DW [8]. These parabens were tested individually and in combination as a MIX formulation, consisting of MP, PP, and BP, each at 0.15 µg/L. The impact of parabens on biofilms was evaluated at this concentration, representative of the range detected in DW [8]. Stock solutions were prepared according to each compound’s characteristic, due to their limited water solubility. MP stock solutions were prepared in ultrapure sterile water to minimize potential chemical interference [16], whereas PP and BP stock solutions were prepared using Ac at a final concentration of 0.005% (*v*/*v*). Accordingly, PP- and BP- and MIX-exposed samples were compared with the respective acetone control to exclude potential effects associated with the solvent. The physicochemical properties of the selected parabens are presented in Table 3.

### 4.2. Bacteria and Culture Conditions

*Acinetobacter calcoaceticus* and *Stenotrophomonas maltophilia*, two Gram-negative bacteria previously isolated from DW, were selected as model DW bacteria [45]. These bacteria are known biofilm-forming microorganisms that proliferate within DWDS, raising global concern due to their association with healthcare-associated infections, particularly in immunocompromised individuals, and their notable antibiotic resistance profiles [45,46,47,48,49]. *A. calcoaceticus* is recognized as an emerging opportunistic pathogen associated with healthcare-related infections and with antibiotic resistance patterns comparable to those of *Acinetobacter baumannii* [46,47]. This species is also known for its strong coaggregation capacity, playing a bridging role in DW biofilm formation [48]. *S. maltophilia* is also an emerging opportunistic pathogen and has been frequently detected in DW systems [49].

These strains were cryopreserved at −80 °C, in aliquots of R2A medium growth with 30% (*v*/*v*) of glycerol. Both bacteria were grown overnight at 25 °C and under agitation (160 rpm) in R2A broth medium prepared as described by Gomes et al. [50].

The number of cells in suspension was adjusted to 1 × 10^8^ CFU/mL for both bacteria. To achieve this, bacteria were harvested from overnight bacteria suspension by centrifugation (Eppendorf centrifuge 5810R, Hamburg, Germany) at 3772× *g* for 10 min and resuspended in fresh R2A broth medium. For the assessment of the virulence factors (Section 4.4), bacteria were washed with STW composed by 100 mg/L NaHCO_3_ (Fisher Scientific, Hampshire, UK), 13 mg/L MgSO_4_.7H_2_O (Merck, Darmstadt, Germany), 0.7 mg/L K_2_HPO_4_ (Aplichem Panreac, Darmstadt, Germany), 0.3 mg/L KH_2_PO_4_ (Chem-Lab, Zedelgem, Belgium), 0.01 mg/L (NH_4_)_2_SO_4_ (Labkem, Barcelona, Spain), 0.01 mg/L NaCl (VWR, Leuven, Belgium), 0.001 mg/L FeSO_4_.7H_2_O (VWR PROLABO, Leuven, Belgium), 1 mg/L NaNO_3_ (Labkem, Barcelona, Spain), 27 mg/L CaSO_4_ (Labkem, Barcelona, Spain), 1 mg/L humic acids (Sigma-Aldrich, Steinheim, Germany).

### 4.3. Biofilm Formation and Exposure to Parabens

Single-species biofilms were formed in the presence and absence of parabens (MP, BP, PP and MIX) at a concentration of 0.15 µg/L over 26 days in PVC (1 cm × 1 cm) coupons from Neves & Neves (Trofa, Portugal) inside 48-well microtiter following the approach described by Gomes et al. [50]. By using single-species biofilms of DW bacteria, system complexity and experimental variability were reduced, enabling a more controlled assessment of the direct effects of paraben exposure on bacterial phenotypic responses.

To clean and sterilize coupons for biofilm formation, coupons were immersed in a solution of a commercial detergent (Sonasol Pril, Henkel Ibérica S.A., Barcelona, Spain) in distilled water for 30 min twice. Afterwards, the coupons were rinsed in distilled water and immersed in ethanol at 70% for 30 min. Then, coupons were rinsed with distilled sterile water, dried overnight at 60 °C, and exposed to UV for 30 min each side.

For biofilm formation, 1 mL of bacterial suspension adjusted to 1 × 10^8^ CFU/mL was added in each well. Microtiter plates were incubated for 24 h at 25 °C with agitation (160 rpm). Following incubation, the colonized coupons were rinsed with 1 mL per well of STW to remove loosely attached and non-adherent cells [51]. Biofilms were then exposed to the respective parabens prepared in R2A medium. After 12 h, coupons were washed again with STW and re-exposed to the corresponding paraben solutions prepared in STW to simulate the low-nutrient conditions typically found in DW. Parabens solutions were renewed every 2 days to maintain continuous exposure at a constant concentration throughout the 26-day experimental period. Biofilms exposed only to STW or to Ac at 0.005% (*v*/*v*) were used as negative and solvent controls, respectively. The entire biofilm formation setup was performed three independent times.

After exposure, the colonized coupons with parabens-exposed and non-exposed biofilms were inserted in a 15 mL centrifuge tube containing 5 mL of sterile saline water and vigorously vortexed for 2 min (to ensure the complete removal of adhered bacteria and the dissociation of possible bacterial aggregates without compromising bacterial viability). This biofilm suspension was used for further culturability assessment by plating the biofilm suspensions through appropriate serial dilutions in R2A agar using the drop plate method, and cellular density quantification through 4′,6-diamidino-2-phenylindole (DAPI) staining as described by Pereira et al. [5].

### 4.4. Virulence Factors Production

The production of virulence factors of both bacteria from planktonic cultures and from 26-day old biofilms grown on PVC coupons with the presence and absence of parabens solutions (MP, PP, BP, and MIX) was evaluated. For that, bacterial cells from single-species biofilms were previously cryopreserved at −80 °C, in aliquots of R2A medium growth with 30% (*v*/*v*) of glycerol to be used for further assays. Cryopreserved *A. calcoaceticus* and *S. maltophilia* (from biofilms) were grown overnight at 25 °C and under agitation (160 rpm) in R2A broth medium as described in Section 4.2. Then, the bacterial cell density was adjusted to 10^8^ CFU/mL in STW for further evaluation of motility, enzymes production, and siderophores production.

#### 4.4.1. Bacterial Motility

Bacterial swimming, swarming, and twitching motilities were evaluated using agar media with different concentrations, following the methodology described by Pereira et al. [5]. For each assay, 15 µL of bacterial suspension was inoculated at the center of agar plates containing tryptone (Merck, Darmstadt, Germany) at 10 g/L, NaCl (VWR, Leuven, Belgium) at 2.5 g/L, and agar (VWR, Leuven, Belgium) at 3 g/L, 7 g/L, and 15 g/L for swimming motility, swarming motility, and twitching motility assays, respectively. Then, the plates were incubated at 25 °C for 72 h and the colony growth halo was measured after 24, 48 and 72 h of incubation using three independent assays performed in triplicate.

#### 4.4.2. Enzymes Production

The production of extracellular enzymes by planktonic cells and biofilm-grown bacteria exposed or not exposed to parabens for 26 days was evaluated by assessing gelatinase, protease, and lipase activities. Gelatinase production was determined using a phenotypic assay adapted from Lopes et al. [52]. Briefly, 10 µL of bacterial suspension was spotted onto gelatin agar plates composed of peptone (5 g/L; Merck, Darmstadt, Germany), yeast extract (3 g/L; Merck, Darmstadt, Germany), gelatin (30 g/L; Oxoid, Hampshire, UK), and agar (15 g/L; VWR, Leuven, Belgium) at pH 7.

Plates were incubated at 25 °C for 48 h and subsequently flooded with a saturated ammonium sulfate solution (2.84 M; VWR, Leuven, Belgium). Gelatinase production was identified by the formation of a transparent halo surrounding bacterial growth due to gelatin precipitation, and both the clearance zone and growth halo diameters were measured at the end of incubation.

Protease production was assessed by inoculating 10 µL of bacterial suspension onto plate count agar (PCA; VWR, Leuven, Belgium) supplemented with 10 g/L skim milk powder (Merck, Darmstadt, Germany), placing the inoculum at three distinct positions on each plate. Plates were incubated at 37 °C for 72 h, and protease production was indicated by the presence of clear zones around the colonies, which were measured after incubation [53].

Lipase production was evaluated on Luria–Bertani (LB) agar (Sigma-Aldrich, Steinheim, Germany) supplemented with CaCl_2_ (2.0 g/L; Merck, Darmstadt, Germany), agar (15.0 g/L; VWR, Leuven, Belgium), and Tween-80 (10 g/L; VWR, Washington, DC, USA). A volume of 15 µL of bacterial suspension was applied to each plate, and lipase production was determined after 72 h of incubation by measuring the diameter of the clear halo surrounding the colonies. A positive reaction was identified by the presence of a light-yellow transparent zone around bacterial growth [54]. The results were presented as the difference between clearance zone and colony diameter. For the evaluation of enzymes production three independent assays performed in triplicate.

#### 4.4.3. Siderophores Production

Differences in siderophore production were evaluated for both planktonic and biofilm cells exposed or not exposed to parabens. Siderophore production was assessed using chrome azurol S (CAS) agar plates prepared with R2A medium (Merck, Darmstadt, Germany). The CAS–iron dye solution was prepared by dissolving 60.7 mg of chrome azurol S (Fluka, Buchs, Switzerland) in 50 mL of distilled water, followed by the addition of 10 mL of an iron (III) solution (VWR, Darmstadt, Germany). This mixture was then combined with 40 mL of a hexadecyltrimethylammonium bromide (HDTMA) solution containing 72.4 mg of HDTMA (Merck, Darmstadt, Germany) dissolved in distilled water. Briefly, 25 mL of a sterilized solution of CAS-iron dye prepared according to Neilands [55] was added to 250 mL R2A agar. A volume of 20 µL of bacterial suspension was placed in the center of each plate of CAS agar. Plates were incubated for 48 h at 25 °C and siderophores production was evaluated by the formation of an orange halo that was measured (mm) after the incubation period. The results were presented as the difference between orange halo and colony diameter. At least three independent assays were performed in triplicate.

#### 4.4.4. Biofilm Development After Exposure to Parabens

The ability of isolated bacterial cells from biofilms exposed to parabens at 0.15 µg/L for 26 days to form new biofilms was evaluated in terms of culturability and total biomass production. For that, cryopreserved bacterial cells from 26-day-old *A. calcoaceticus* and *S. maltophilia* biofilms non-exposed and exposed to the selected parabens (MP, PP, BP, and MIX) were grown overnight in R2A at 25 °C and 160 rpm of agitation as described in Section 4.2. Cell concentration was adjusted to 1 × 10^8^ CFU/mL in the same fresh medium (R2A). Then, bacterial biofilms were formed in sterile 96-well polystyrene microtiter plates (VWR, West Chester, PA, USA), according to Stepanović et al. [56]. Each well was filled with 200 μL of bacterial suspension (1 × 10^8^ CFU/mL in R2A broth). At least six wells were evaluated for each condition, and three independent experiments were performed. Negative control wells were filled with sterile R2A broth. After 24 h incubation at 25 °C under 160 rpm, biofilms were washed with NaCl at 8.5 g/L to remove all non-adherent and weakly adhered bacteria. Then, biofilm production was assessed in terms of CFU enumeration in R2A agar (Merck, Darmstadt, Germany), and by CV (Merck, Darmstadt, Germany) staining as described by Gomes et al. [50].

### 4.5. Tolerance to Antimicrobials

To evaluate the long-term effects of parabens exposure on changing bacterial susceptibility to antibiotics, both planktonic bacteria were exposed to MP, which was selected as a representative of the paraben class due to its widespread use in personal care products worldwide [57]. For that, both *A. calcoaceticus* and *S. maltophilia* were grown on R2A agar without MP and supplemented with MP at 0.15 µg/L for 10 weeks, followed by an additional 10-week growth period on fresh R2A agar in the absence of MP to assess the persistence of exposure-related effects. During this time, every week both bacteria were passed to a new fresh agar plate. The susceptibility to antibiotics was evaluated in the 5th and 10th weeks by the disc diffusion susceptibility methods according to the Clinical and Laboratory Standards Institute (CLSI) [58]. CEF at 30 µg; levofloxacin (LEV) at 5 μg; MINO at 30 µg and TMP-SMX at 1.25/23.75 μg were the selected antibiotics, according to the CLSI guidelines [58]. For that, bacteria were adjusted to 0.135 at 600 nm to match 0.5 MacFarland and spread on Mueller–Hinton agar—MHA (Merck, Darmstadt, Germany)—for all conditions. Then, antibiotic-containing discs (Oxoid, UK) were inserted on the top of the MHA plate and further incubated at 37 °C for 24 h before measuring the diameter of growth inhibition. Each condition was tested in duplicate with three independent experiments. Inhibition halos (cm) are presented as average ± standard deviation.

### 4.6. Statistical Analysis

The experimental data were analyzed using the statistical program GraphPad Prism 8.0 for Windows 10 (GraphPad Software, La Jolla, CA, USA). The mean and standard deviations within samples were calculated using descriptive statistics for all cases based on a minimum of three independent assays, with duplicates. The normal distribution of data was checked using D’Agostino and Pearson omnibus normality test. Statistical calculations were based on a confidence level of ≥95% (*p* < 0.05 was considered a statistically significant difference). For more than three groups statistical analysis was done using ANOVA, followed by Tukey’s multiple comparisons test. Unpaired *t*-tests were performed to compare two samples under distinct conditions.

## 5. Conclusions

This study demonstrates that long-term exposure (26 days) to environmentally relevant concentrations of parabens found in DW can alter the phenotypic bacterial behavior of DW biofilm bacteria in a species- and compound-dependent manner. Individual solutions of PP, BP, and parabens in combination (MIX treatment) caused the most pronounced effects, promoting biofilm regrowth and enhancing virulence-associated traits, such as motility and lipase production. This highlights that increasing alkyl chain length and exposure to complex paraben mixtures are more likely to induce microbial modifications. Additionally, prolonged MP exposure (10 weeks) increased antimicrobial tolerance in *A. calcoaceticus*. These findings suggest that parabens in DW may act as selective pressures shaping bacterial behavior, with potential implications for DW safety and public health. This study underscores the need to integrate microbial endpoints into chemical risk assessment and to consider mixture effects when evaluating the safety of emerging contaminants in DW.

## Figures and Tables

**Table 1:** Colony growth halo (mm) of motility, extracellular enzymes, and siderophores production of 26-day-old biofilm-derived *A. calcoaceticus* cells formed on PVC exposed and non-exposed to parabens (0.15 µg/L).

Virulence Factors	Control Biofilm (STW)	Control Biofilm (Ac)	MP	PP	BP	MIX
Swimming	63.4 ± 3.2 *^a^#	55.1 ± 3.6 #	43.9 ± 5.9 ^a^	48.5 ± 14.5	51.4 ± 8.8	57.2 ± 2.4
Swarming	30.8 ± 6.1 *^b^#	12.3 ± 0.7 #	12.2 ± 0.4 ^b^	13.2 ± 1.4	12.3 ± 0.6	12.8 ± 0.2
Twitching	14.0 ± 0.2 *^c^	11.6 ± 0.4	12.0 ± 0.0 ^c^	12.8 ± 1.5	13.9 ± 3.9	12.6 ± 1.3
Protease	38.7 ± 2.2 *	38.6 ± 3.8	36.0 ± 0.3	42.0 ± 5.6	38.8 ± 4.0	43.7 ± 3.5
Gelatinase	24.4 ± 4.1 *	24.0 ± 3.3	23.0 ± 3.4	21.0 ± 2.9	22.8 ± 3.0	25.3 ± 3.5
Lipase	18.2 ± 2.7	16.5 ± 1.2	16.6 ± 1.8	16.9 ± 1.6	18.1 ± 1.0	16.7 ± 0.9
Siderophores	17.0 ± 5.0 *	17.2 ± 4.7	22.0 ± 2.4	22.0 ± 2.0	20.3 ± 1.0	18.8 ± 3.3

The average diameter of the initial drop was 7.1 ± 0.5 mm. *—Planktonic (Supplementary Material Table S1) and biofilm cells samples were statistically different; #—Control (STW) is statistically different from control (Ac); ^a, b, c^—samples were statistically different from the respective control (ANOVA, post hoc Tukey’s test, *p* < 0.05).

**Table 2:** Colony growth halo (mm) of motility, extracellular enzymes, and siderophores production of 26-day-old biofilm-derived *S. maltophilia* cells formed on PVC exposed and non-exposed to parabens (0.15 µg/L).

Virulence Factors	Control Biofilm (STW)	Control Biofilm (Ac)	MP	PP	BP	MIX
Swimming	64.3 ± 3.6 *^b^#	38.5 ± 9.7 ^f,g^#	22.0 ± 2.1 ^b^	41.7 ± 4.0	62.2 ± 3.7 ^f^	86.0 ± 0.0 ^g^
Swarming	26.3 ± 2.7 *^c^#	11.7 ± 0.6 ^h,i^#	11.0 ± 0.5 ^c^	12.1 ± 7.0	42.8 ± 4.2 ^h^	62 ± 23.3 ^i^
Twitching	12.7 ± 0.7	12.1 ± 2.0	11.0 ± 1.0	13.6 ± 2.0	12.3 ± 0.5	12 ± 0.3
Protease	35.2 ± 0.2 *	40.2 ± 5.8	34.0 ± 10	37.9 ± 3.0	35.8 ± 2.1	34.0 ± 2.4
Gelatinase	19.5 ± 2.8	18.8 ± 1.3	18.0 ± 1.5	19.3 ± 1.0	20.1 ± 2.2	18.0 ± 1.5
Lipase	32.0 ± 0.5 *^a^#	18.0 ± 0.5 ^d,e^#	18.8 ± 1.3 ^a^	18.8 ± 1.3	27.8 ± 1.6 ^d^	31.1 ± 0.5 ^e^
Siderophores	22.2 ± 4.5 *	18.8 ± 1.0	19.0 ± 1.1	19.4 ± 9.0	22.0 ± 2.8	22.0 ± 1.2

The average diameter of the initial drop was 7.1 ± 0.5 mm. *—Planktonic (Supplementary Material Table S1) and biofilm cells samples were statistically different; #—Control (STW) is statistically different from control (Ac); ^a, b, c, d, e, f, g, h, i^—samples were statistically different from the respective control (ANOVA, post hoc Tukey’s test, *p* < 0.05).

**Table 3:** Physicochemical properties of the selected parabens.

Parabens	MP	PP	BP
Chemical structure	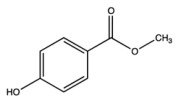	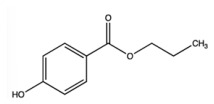	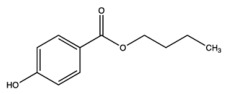
Molecular formula	C_8_H_8_O_3_	C_10_H_12_O_3_	C_11_H_14_O_3_
Molecular weight (g/mol)	152.15	180.20	194.23
Boiling point (°C)	270–280	294–295	156–157
Melting point (°C)	131	97	68–69
log acid dissociation constant (pKa)	8.17	8.35	8.37
log octanol-water partition coefficient (log K_OW_)	1.96	3.04	3.57
Vapor pressure at 25 °C (mm Hg)	2.37 × 10^−4^	3.07 × 10^−4^	2.51 × 10^−4^
Henry’s Law constant at 25 °C (atm m^3^/mol)	2.23 × 10^−9^	4.25 × 10^−9^	6.00 × 10^−9^

Physicochemical properties of parabens are available at Safety Data Sheets of compounds in Supelco (www.sigmaaldrich.com, accessed on 5 May 2026).

## Data Availability

The datasets used in the present study will be made available on reasonable request.

## Supplementary Materials

The following supporting information can be downloaded at https://www.mdpi.com/article/10.3390/antibiotics15060565/s1, Figure S1: Culturability of biofilm cells (log CFU/cm^2^) and cellular density (log cells/cm^2^) of 26-day-old (■ *A. calcoaceticus* ■ *S. maltophilia*) single-species biofilms formed on PVC exposed and non-exposed to parabens (0.15 µg/L).; Table S1: Colony growth halo (mm) of motility, extracellular enzymes and siderophores production of planktonic *A. calcoaceticus* and *S. maltophilia*.

## Abbreviations

The following abbreviations are used in this manuscript:

Ac	acetone
ARGs	antibiotic resistance genes
BP	butylparaben
CEF	ceftazidime
CFU	colony-forming units
CV	crystal violet
DW	drinking water
DWDS	drinking water distribution systems
LEV	levofloxacin
MHA	Mueller-Hinton agar
MINO	minocycline
MP	methylparaben
PP	propylparaben
STW	synthetic tap water
TMP-SMX	trimethoprim-sulfamethoxazole
USA	United States of America
