# Supplementary material for: Parabens at Environmental Levels Modulate Virulence and Antimicrobial Tolerance of Exposed Biofilm Cells
Source: Antibiotics (Basel). 2026 Jun 1;15(6):565. doi: 10.3390/antibiotics15060565 (PMC13295724; doi:10.3390/antibiotics15060565)
Supplement: Supplementary file 1 [file antibiotics-15-00565-s001.zip › antibiotics-4329286-supplementary.pdf]

Supplementary Material

Characterization of 26-day-old single-species biofilms

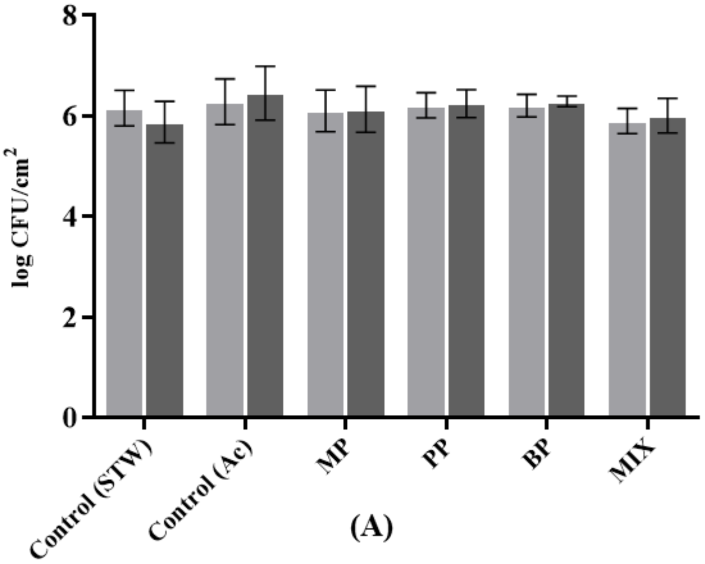

(A)

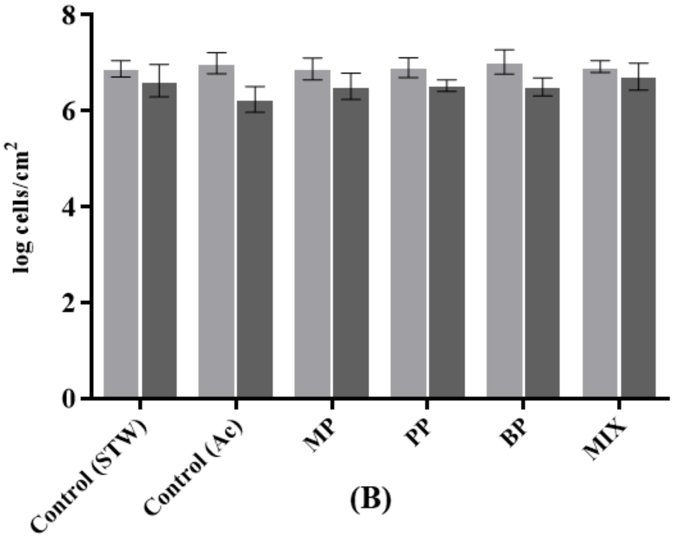

(B)

Figure S1 - Culturability of biofilm cells (log CFU/cm^2) and cellular density (log cells/cm^2) of 26-day-old (■ *A. calcoaceticus* ■ *S. maltophilia*) single-species biofilms formed on PVC exposed and non-exposed to parabens (0.15 µg/L).

Table S1 - Colony growth halo (mm) of motility, extracellular enzymes and siderophores production of planktonic *A. calcoaceticus* and *S. maltophilia.*

**Virulence factors**	***A. calcoaceticus***	***S. maltophilia***
**Swimming**	11.8 ± 3.2[a]	20.8 ± 3.0[a]
**Swarming**	9.6 ± 0.5	10.0 ± 1.7
**Twitching**	12.0 ± 1.7	12.2 ± 1.1
**Protease**	14.5 ± 3.1[b]	41.5 ± 2.9[b]
**Gelatinase**	0[c]	20.5 ± 2.0[c]
**Lipase**	13.1 ± 2.5	12.7 ± 0.4
**Siderophores**	0[d]	13.2 ± 1.5[d]

[a, b, c, d] - samples were statistically different between them (ANOVA, post-hoc Tukey's test, $P < 0.05$).
